# NKTs are involved in the role that natural lipids play in the sensitisation to the major allergen of brazil nuts, Ber e 1

**DOI:** 10.1186/2045-7022-1-S1-O12

**Published:** 2011-08-12

**Authors:** Luciana Mirotti, Maria Leite-de-Moraes, Morntchilo Russo, Stella Cochranea, Marcos Alcocer

**Affiliations:** 1 University of Sao Paolo, Immunology Department, Sao Paolo, Brazil; 2 Hospital Necker, Paris, France; 3 University of Sao Paulo, Sao Paulo, Brazil; 4 Unilever, Bedford, UK; 5 University of Nottingham, Nottingham, UK

## 

The prevalence of food allergy is increasing in westernized countries, affecting around 4% of the population. Although innumerous proteins are encountered in normal diets, only few protein families are commonly implicated as food allergens. In order to be able to predict the allergenicity of food proteins, recent studies focused on intrinsic features of these few families of allergens; however, it is known that extrinsic factors can play a role in allergic processes. The allergenicity of Ber e 1, the major allergen from Brazil nuts, is well established. It has been suggested that natural lipids from Brazil nuts play a role in the development of an immune response towards Ber e 1. Therefore the present study aimed to characterize the humoral response induced by recombinant (r)Ber e 1 in the presence of different classes of lipids, and to investigate the role played by natural lipids in the immune sensitization.. BALB/c mice were sensitised i.p. with rBer e 1 alone or in the presence of different natural lipid fractions. It was found that rBer e 1 alone did not induce antibody production, even in the presence of alum. Only one specific fraction of Brazil nut lipids (SPC C), was able to induce a Th2-type humoral response, with the presence of Ber-specific anaphylactic antibodies, high levels of Ber-specific IgG1, and low levels of Ber-specific IgG2a. In order to test the hypothesis that NKT cells may be involved in the response via CD1 receptor the sensitisation protocol with rBer e 1 and SPC C fraction was tested in mice lacking these cells (JALPHA18 KO mice). These animals presented significantly lower titers of Ber-specific anaphylactic antibodies (Ber-specific IgG1and total IgE) than sensitised wild type mice, indicating that one of the pathways by which lipids triggered an immune response involves NKT cells. In vitro assay of TLRs (2 to 9) activation showed that SPC C fraction was not able to activate these receptors. In conclusion, our recent results corroborate the idea that lipids from Brazil nuts are essential for the development of a Th2-type humoral response to rBer e 1 and suggest that the immune response induced by lipids might involve NKT cells.

